# Community-onset sepsis and its public health burden: protocol of a systematic review

**DOI:** 10.1186/s13643-015-0103-6

**Published:** 2015-09-23

**Authors:** Alexander Tsertsvadze, Pam Royle, Noel McCarthy

**Affiliations:** Communicable Disease Control Epidemiology and Evidence; Populations, Evidence and Technologies; Division of Health Sciences; Warwick Medical School, University of Warwick, Coventry, CV4 7AL UK

**Keywords:** Community-onset sepsis, Risk factors, Incidence of sepsis or severe sepsis

## Abstract

**Background:**

Sepsis is a life-threatening condition and major contributor of public health and economic burden in the industrialised world. The heterogeneity, absence of more specific definition, and difficulties in accurate diagnosis lead to great variability in the estimates of sepsis incidence. There has been uncertainty regarding the incidence and risk factors attributable to community-onset as opposed to hospital-acquired sepsis. Community-onset sepsis has distinct host characteristics, risk factors, pathogens, and prognosis. A systematic assessment of recent evidence is warranted in light of secular changes in epidemiology, pathogens, and the uncertainties around the incidence and risk factors of community-onset sepsis.

This protocol describes a systematic review which aims to synthesise the recent empirical evidence on the incidence and risk factors of community-onset sepsis, severe sepsis, and septic shock in high-income countries.

**Methods/Design:**

English-language publications of cohort and case-control studies reporting incidence and risk factors of community-onset sepsis will be eligible for inclusion. MEDLINE and Embase databases will be searched from 2002 and onwards. References of relevant publications will be hand-searched. Two reviewers will independently screen titles/abstracts and full texts as well as extract data and appraise the risk of bias of included studies. The data extractions and risk of bias assessments will be cross-checked. Any disagreements will be resolved via consensus.

The data on incidence and risk factors of sepsis will be organised and synthesised in text, tables, and forest plots. The evidence will be pooled given sufficient data and degree of similarity across study populations, exposures, and outcomes. The heterogeneity will be assessed through visual inspection of forest plots, Chi-square-based *p* value, and *I*^2^ statistic. The sources of heterogeneity will be explored via subgroup analysis.

**Discussion:**

Timeliness and accuracy of diagnosis of sepsis are both crucial aspects for improving the patient’s outcome. The findings of this review will be discussed with a view to better inform future recommendations on improving public-facing campaigns, timely presentation, and diagnosis of sepsis in the community. The review will also discuss gaps in evidence and highlight future research and policy-making avenues for improving public health planning.

**Systematic review registration:**

PROSPERO CRD42015023484

## Background

### Context for this review

The UK Department of Health allocated the task to ‘Review the evidence and make recommendations on the scope for a public-facing campaign to raise awareness of Sepsis.’ (https://www.gov.uk/government/publications/phe-remit-letter-2015-to-2016) to Public Health England in the 2015–2016 remit letter to PHE. The University of Warwick was requested to synthesise relevant evidence including estimating the burden of community-onset sepsis and groups affected by community-onset sepsis. This is the motivation for this work.

### Health and economic burden

Sepsis is a complex life-threatening condition characterised by the host’s systemic anti-inflammatory immune response to infection, which may lead to organ damage, organ failure, septic shock, and death [1]. Sepsis with its associated complications remains a major public health and economic burden in the industrialised world [2]. Outcomes of sepsis may have serious short- or long-term consequences such as amputation, damage to organs, or cognitive dysfunction. In the US, treatment of a patient with sepsis may cost up to $50,000, translating to an annual nationwide economic burden of $17 billion [3, 4]. In European studies, the treatment of severe sepsis in 2002 was estimated to be approximately £25,000 [5]. Assuming the incidence of 100,000 new cases per year, the UK’s National Health System (NHS) expenditure for caring these cases would amount to £2.5 billion annually [6].

Data on global incidence of sepsis is scarce but has been growing over the past two decades, with the majority of studies identifying sepsis cases from intensive-care unit (ICU) data [4]. The estimates of sepsis incidence are highly variable. This is likely due to the heterogeneous nature, lack of a uniform definition, and difficulties in the accurate diagnosis of the condition as well as the differences in data sources (e.g. clinical registries, hospital discharge databases, or vital statistics records), periods of follow-up time, and methods of estimation used across studies. Moreover, secular changes and genuine differences in the incidence of sepsis across study populations may have additionally contributed to the observed variability. For example, one systematic review [7] reported the following ranges of annual incidence for sepsis (149–240 per 100,000 population) and severe sepsis (56–91 per 100,000 population). Another more recent systematic review of 33 epidemiologic studies conducted in 15 high-income countries [8] estimated and reported an average incidence of 427 (95 % CI 281, 648) for sepsis and 331 (95 % CI 207, 530) for severe sepsis cases per 100,000 person-years.

Large nationwide cohort studies conducted in five high-income countries (the USA, the UK, France, Australia, and New Zealand) in 1995–2002 showed a wide variation in the annual incidence of severe sepsis, ranging from 51 [9] to 300 [3] cases per 100,000 population [4, 3, 9–11]. The study by Padkin et al. [9] which reviewed data from 91 ICUs across England, Wales, and Northern Ireland reported an annual incidence of 51 cases of severe sepsis per 100,000 persons. These findings are in agreement with another UK-based cohort study which reported an incidence of 66 cases per 100,000 per population [12]. More recent studies conducted in Europe reported lower incidence rates of 38 [13] and 25 cases [14] per 100,000 population. Although variable, the results of these nationwide cohort studies nonetheless all indicate that severe sepsis is a common disorder [2].

Recent estimates of case-fatality rates for sepsis ranged from 14.7 % [15] to 28.6 % [3] in the US-based studies [3, 4, 15] and from 35.0 % [11] to 53.6 % [16] in European studies [11, 16, 10, 9, 5]. Almost one third of all ICU admissions in the UK are related to sepsis and about half of these patients die [9]. In their study [17], McPherson and colleagues reported that one in 20 deaths in England in 2001–2010 was associated with sepsis. These figures may underestimate the true mortality rate. Sepsis is often underreported as a cause of death because of the absence of the sepsis-specific International Classification of Diseases, 10th Revision (ICD-10) codes. Therefore, the sepsis-related deaths are often coded as deaths caused by kidney infection, pneumonia, influenza, or meningitis [17].

Over the past two decades, there has been accumulation of empirical evidence showing a gradual increase (8–13 % per year) in the incidence of sepsis in high-income countries (e.g. the UK, Australia, Croatia) [18], especially in the USA [4, 12, 19]. For example, one UK-based cohort study found an increase in annual incidence of severe sepsis from 46 (in 1996) to 66 cases per 100,000 per population between 1996 and 2003 [12]. In contrast to the incidence data, the mortality (i.e. case-fatality rate) after sepsis in high-income countries has been decreasing [15, 20]. For example, one US-based study reported the decrease in case-fatality rate of sepsis from 27.8 % (1979–1984) to 17.9 % (1995–2000) [4]. Similarly, in their ICU-based study [12], Harrison et al. showed a significant decrease in mortality rate from 48.3 % (in 1996) to 44.7 % (in 2004). The observed trends of rising incidence could be due to increased proportions of high-risk population subgroups (e.g. elderly, type-II diabetes, antibiotic resistance, cancer), improvements in methods of detection [21, 22], and the falling mortality rates due to identification of less severe forms of sepsis (in light of the improved methods of detection) [21, 22] and/or advancements in the treatment of sepsis [23, 12, 22]. In spite of the reduced case-fatality rates, the total annual number of sepsis-related deaths has been rising, perhaps owing to the increased incidence of sepsis [18]. Over the last two decades, Gram-positive bacteria have superseded Gram-negative bacteria as the most common cause of sepsis as further evidence of the changing biology of this condition [18].

### Definition and diagnosis

Sepsis treatment is time-critical, which necessitates timely diagnosis and rapid provision of appropriate management options. However, this is made difficult owing to the heterogeneity of this condition, often characterised by non-specific clinical features. The current definition of ‘sepsis’ which was introduced in 1991 [24] encompasses the presence of infection and more than one of the Systemic Inflammatory Response Syndrome (SIRS) criteria including the following: a) body temperature [>38 or <36 °C], b) heart rate [>90 beats/min], c) hyper-ventilation [respiratory rate >20 breaths/min or PaCo2 < 32 mmHg], and d) white blood cell count [>12,000 cells/μL or <4000 cells/μL]. According to this definition, ‘sepsis’ with organ dysfunction and ‘sepsis’ with acute circulatory failure with arterial hypotension have been termed as ‘severe sepsis and ‘septic shock’, respectively [1]. Although the joint presence of infection and SIRS criteria has been widely adopted, their utility as a diagnostic tool for identifying sepsis has been recognised to be limited owing to high sensitivity and low specificity of these criteria (i.e. they may manifest in the absence of infection, in patients with severe trauma, burns, and other inflammatory disorders such as pancreatitis) [25, 18].

In 2001, the American College of Chest Physicians (ACCP) and the Society of Critical Care Medicine (SCCM) convened a consensus conference and updated the definition of sepsis by expanding the list of markers potentially related to sepsis (e.g. inflammatory response, hemodynamic, organ dysfunction, and tissue perfusion parameters) [1]. One important outcome of this conference was the introduction of a ‘Predisposition, Infection, Response, and Organ Dysfunction’ (PIRO) system for staging sepsis. For the purpose of improving the diagnosis of sepsis, some authors suggested that the original definition of sepsis should additionally incorporate an evidence of organ dysfunction which is a more specific sign to sepsis or severe sepsis [21, 25]. The original definition adopted in 1991 [24] is still widely used. The difficulties in case definition and variations in the definitions used substantially complicate the comparison and synthesis of findings across studies.

### Risk factors and high-risk population subgroups

Several studies examined age, sex, and race disparities for developing sepsis and demonstrated that men compared to women are more likely to have sepsis or severe sepsis [4, 9, 11, 10, 26]. Similarly, African-Americans are at higher risk for developing severe sepsis compared to Caucasians (adjusted relative risk range 1.40–1.89) [4, 27, 28]. Moreover, older age [19, 29] and certain chronic medical conditions (HIV, alcohol abuse, cancer, lung/kidney disease, myocardial infarction, diabetes, stroke, deep vein thrombosis, coronary artery disease, hypertension) [29, 2, 30, 26] have also been shown to be associated with a significantly greater risk for sepsis. In their study, Hall et al. observed a 30-fold increase in the incidence rate of sepsis or septicaemia amongst people >85 years vs. those ≤65 years (271.2 per 10,000 vs. 9.5 per 10,000) [19]. In one US-based nationwide cohort study, the risk of sepsis in cancer patients was almost 10 times as high compared to the US general population without cancer (age- and sex- adjusted RR = 9.77, 95 % CI 9.67, 9.88) [30].

### Community-onset and hospital-acquired sepsis

Traditionally, sepsis has been classified into community-onset and hospital-acquired (i.e. nosocomial) infection, depending on the place of the infection’s acquisition [31, 32]. The two contexts of sepsis acquisition differ in terms of the host characteristics (e.g. demographics, risk profile, resistance patterns), pathogens, and outcomes [33–36]. For example, in their study, Hoenigl and colleagues observed a significantly higher 30-day (20.75 vs. 11.20 %, *p* = 0 · 001) and 90-day (26.83 vs. 12.63 %, *p* < 0 · 001) mortality rates in people with hospital-acquired vs. community-onset infection [34].

More recently, with increasing number of sepsis cases associated with outpatient treatment that takes place in communities (e.g. nursing homes, dialysis, long-term home care facilities), a new category of healthcare-associated sepsis has been recognised and introduced [37, 38]. According to the definition of healthcare-associated sepsis, the patient had to have received a medical care in the community/outpatient setting (e.g. intravenous therapy, wound care) 30 days before the bloodstream infection, hospitalisation in acute care hospital 90 days before the bloodstream infection, attendance of hospital or haemodialysis clinic, or residence in a nursing home or a long-term care facility [37]. Although nosocomial and healthcare-associated sepsis are similar with respect to source of infection, type of pathogens, susceptibility, and the outcome, emerging empirical evidence has shown them to be two distinct entities. Therefore, community-onset sepsis has been further divided into healthcare-associated and community-acquired sepsis [37–39, 34, 35].

The definition of community-onset sepsis has not been consistent in the literature [39]. The most often used and widely accepted definition specifies community-onset sepsis as one that manifests (positive blood culture and systemic inflammatory response syndrome criteria) before or within 48 h after hospital admission [31, 37, 34, 40, 36, 41]. According to this definition, a nosocomial (hospital-acquired) infection is one that manifests more than 48 h after hospitalisation [37, 34, 40, 36, 41].

One important gap in the sepsis literature is the scarcity of evidence on the incidence of sepsis as opposed to severe sepsis for which evidence is more abundant [18]. Another limitation is that the majority of studies have used hospital discharge databases which do not allow to distinguish the findings between community-onset and hospital-acquired sepsis, which as noted above are different in population distribution and outcome. The evidence on incidence and risk factors of community-onset sepsis has not been systematically reviewed, an evidence-base gap of particular importance in planning for public-facing interventions.

To address these gaps, this systematic review will identify, appraise, and synthesise the empirical evidence on the incidence and risk factors of community-onset sepsis, severe sepsis, and septic shock.

### Research question and aims of the systematic review

The aim of this review will be to systematically identify, appraise, and synthesise the recent evidence on incidence of community-onset sepsis, severe sepsis, or septic shock in countries of the Western industrialised world (North America, Australasia, and North/Western Europe). The review focus will be limited to more recent evidence from high-income countries (more relevant to the UK practice) given the documented longitudinal changes in the incidence, outcome, and implicated pathogens in sepsis [18] and modifications in the definition of sepsis that have taken place in 1991 [24] and 2001 [1]. Therefore, only studies reporting the evidence on data collected in 2002 or onwards will be sought.

To contribute to the aim of supporting PHE responsibilities to evaluate the evidence base for a public-facing sepsis campaign, the specific review objectives will be the following:

- To catalogue and map in a tabular fashion the relevant literature according to socio-demographic characteristics and clinical risk factors in relation to incidence of community-onset (e.g. healthcare-associated, community-acquired) sepsis, severe sepsis, and/or septic shock
- To document overall and stratum-specific (by socio-demographic characteristics, clinical risk factors) incidence of community-onset (e.g. healthcare-associated, community-acquired) sepsis, severe sepsis, and/or septic shock
- To compile and synthesise evidence on specific socio-demographic, clinical, or laboratory characteristics as potential risk factors for community-onset (e.g. healthcare-associated, community-acquired) sepsis, severe sepsis, and/or septic shock

## Methods/Design

This systematic review protocol will be reported according to recommendations from the Preferred Reporting Items for Systematic Review and Meta-analysis Protocols (PRISMA-P) 2015 statement [42].

### Study eligibility criteria (primary studies)

#### Inclusion criteria

##### Study design

Longitudinal prospective or retrospective cohort studies; case-control studies.

##### Study setting

Population- or hospital-based studies considering community-onset cases separately; studies conducted in North America, Australasia, and North/Western Europe.

##### Population

Community dwellers, hospitalised patients (male of female) from a defined population of any age (except for neonates) and health state with or without community-onset sepsis at study baseline. The use of relevant ICD-9/10 codes [3, 4, 43] and established criteria for the diagnoses of sepsis (e.g. the presence of infectious pathogen or bloodstream infection plus two or more SIRS criteria as a direct response to the infection), severe sepsis (e.g. sepsis complicated by organ dysfunction), and septic shock (e.g. sepsis-induced acute circulatory failure associated with persistent arterial hypotension) [24, 1, 33, 44]. will be used to determine inclusion. Those with community-onset sepsis will be eligible for inclusion regardless whether they have healthcare-associated (HCA; e.g. people receiving outpatient treatments such as dialysis) or community-acquired (CA) sepsis.

##### Intervention/exposure 1

Any subgroup, patient characteristic, or clinical parameter (e.g. age, sex, comorbidity, heart rate, body temperature, altered mental status, white blood cell count, creatinine, organ dysfunction score) evaluated for association with risk of sepsis, severe sepsis or septic shock.

##### Comparator/exposure 2

Any subgroup, patient characteristic, or clinical/laboratory parameter used as the reference category to exposures in the exposure 1 group.

##### Outcome

The occurrence of community-onset sepsis, severe sepsis, and/or septic shock. This will allow variations in sepsis definition including studies reporting confirmed bloodstream infections plus SIRS criteria.

##### Outcome measures

Odds, cumulative incidence proportion (risk), incidence rate, and/or hazard rate of sepsis, severe sepsis, and/or septic shock.

##### Measures of association

Odds ratio (OR), risk ratio (RR), risk difference (RD), incidence rate ratio (IRR), and/or hazard ratio.

##### Date of publication

Studies reporting the evidence on data collected in 2002 or onwards.

##### Language of publication

English.

##### Type of publication

Full-text report.

#### Exclusion criteria

##### Study design

Intervention studies (controlled or uncontrolled), prognostic studies (looking at associations between putative prognostic factors and subsequent outcomes or complications such as severe sepsis, septic shock, and/or mortality in participants with sepsis), cross-sectional studies, ecological studies, case series, case reports.

##### Publication type

Abstracts, reviews (systematic or non-systematic), editorials, letters, books, consensus statements, or opinions. Reviews will be excluded as sources of primary data but will be used to identify the original studies contributing the evidence.

##### Publication language

Any other than English. Non-English publications will be excluded due to limited resources. Although the inclusion of non-English studies is likely to cover this topic more comprehensively, to the best of our knowledge, we are not aware of any empirical evidence informing on effects of language bias in systematic reviews of incidence of sepsis.

##### Population

a) Study population with hospital-acquired (nosocomial) sepsis; b) it cannot be determined if the study population presented with community-onset or hospital-acquired sepsis; c) results on populations with community-onset and hospital-acquired sepsis are mixed (not stratified); d) bloodstream infection (BSI) not associated with sepsis/SIRS, severe sepsis (organ dysfunction), or septic shock (circulatory failure and persistent arterial hypotension); e) study population representing a specific subgroup defined by clinical condition (e.g. cancer, coronary heart disease, sepsis/severe sepsis), and f) neonates

##### Outcomes

Studies not reporting incidence/risk of sepsis (in absolute or relative terms), studies reporting only single site infections, or single infecting species, studies reporting only economic evaluation and/or cost-effectiveness outcomes, diagnostic accuracy or prognostic ability of biomarkers, or only mortality (including case-fatality).

### Search strategy and literature sources

We will search Ovid MEDLINE and Ovid Embase from 2002 using a combination of subject headings and keywords for sepsis and related terms combined with terms for epidemiology and related concepts and finally combined this with terms for studies in community-based settings. The Ovid MEDLINE search strategy will also be adapted for Ovid Embase. The searches will be limited to English-language documents, and documents such as comments, letters, editorials, or meeting abstracts will be excluded.

Additionally, we will seek for unpublished literature through the following sources: a) hand search of reference lists of potentially eligible articles, b) relevant websites of organisations dealing with sepsis (International Sepsis Forum, Sepsis Trust UK, Sepsis Alliance, Centre for Disease Control, World Sepsis Day), c) contacting experts/researchers in the field, d) theses database (index to theses), and e) Google Scholar (government or other reports).

We will not search sources of conference proceedings, since they represent abstracts (with no corresponding full texts) which do not provide sufficient information allowing to ascertain and verify a) how sepsis was diagnosed, b) whether study population had community-onset or hospital-acquired sepsis, and c) needed details on incidence and risk factors.

More details on search strategy and sources are provided in Appendix 1.

### Study selection and data management

All bibliographic records (i.e. publications) identified through our searches (electronic or hand-searched) will be compiled and then de-duplicated in a special bibliographic endnote database. Afterwards, two reviewers (AT and NM) using a pre-defined piloted screening form of the eligibility criteria will independently screen all the titles and abstracts of corresponding publications. Any disagreements regarding inclusion or exclusion of any given title/abstract will be discussed and resolved via consensus. Then, the same two reviewers will examine full-text reports of all potentially relevant publications passing the title/abstract level of screening for their eligibility. Any disagreements regarding the eligibility of the full text reports will be discussed and resolved through a consensus agreement or a third party adjudication.

Differentiation of community-onset sepsis (healthcare-associated, community-acquired) from hospital-acquired sepsis will be operationalized by relying on definitions used in individual primary studies indicating the presence of community-onset sepsis. We expect some variation across studies in definitions of community-onset sepsis so that adopting a single pre-specified fixed definition might exclude relevant data. For example, certain authors define community-onset sepsis as one that manifests before hospital admission or within 48 h after hospital admission [34, 40, 36, 41]. Other authors define community-onset sepsis if it manifests within 24–28 h of hospital admission [45, 33, 46]. Some other statements may only suggest the presence of community-onset sepsis ranging from ‘patients hospitalized with sepsis’ to ‘sepsis-related hospitalization’ [47, 28]. Alternatively, other authors may additionally report study exclusion criteria for hospital-acquired sepsis [47].

The study selection process and reasons for exclusion at full-text screening level will be presented in the PRISMA study flow diagram (Appendix 2) [48].

### Data extraction

One of two reviewers (AT and NM) using pre-defined data extraction sheets will extract relevant information from included studies. The extracted data will include information on study (e.g. author name, year of publication, country of conduct, design, study setting, sample size, duration of follow-up, study quality items), potential risk factors (e.g. participant socio-demographic characteristics, comorbidities, health care procedure or intervention, laboratory marker, clinical symptom or parameter), and outcomes (e.g. definition of sepsis and related outcomes, type of pathogen, place and time of sepsis acquisition, type of health care procedure if used as outpatient treatment, the frequency of occurrence measures for sepsis, severe sepsis, and/or septic shock). Any missing statistical parameters of importance (e.g. cumulative incidence proportion, incidence rate, odds, risk ratio, incidence rate ratio, and odds ratio) and variability measures (e.g. 95 % confidence intervals, *p* values) will be calculated, if data permits, or authors of the primary studies will be contacted. All calculated or derived data will be denoted as ‘calculated’ and will be incorporated in the extraction sheets.

The data extracted will be cross-checked. Any disagreements regarding the extracted data will be resolved between the two reviewers or through a consensus agreement or adjudication of a third party, if needed (Appendix 3).

### Quality (risk of bias) assessment

Methodological quality (or risk of bias) of included studies will be appraised by two independent reviewers (AT and NM) using two checklists developed and validated by the Scottish Intercollegiate Guidelines Network (SIGN) separately for cohort [49] and case-control studies [50]. We selected these tools based on the guidance for evidence-based decision making in infectious diseases epidemiology, prevention, and control proposed by Harder and colleagues [51].

Both the cohort (16 items) and case-control (13 items) study checklists address five domains/sources of bias: 1) study research question; 2) participant selection (e.g. sampling bias, differential non-participation, sample attrition/losses to follow-up, incomplete data assessment); 3) information (performance, detection) bias (e.g. outcome and/or exposure measurement and ascertainment, recall bias); 4) confounding, statistical analysis; and 5) an overall assessment of the study (i.e. summary judgement on internal and external validity of study findings). The response to each item can be recorded as ‘yes’, ‘no’, ‘can’t say’, or ‘doesn’t apply’. The overall methodological quality of each included study will be based on the number of satisfied items (response ‘yes’) and will be rated as follows:

- High quality^++^ (≥12 items rated as ‘yes’ for cohort studies and ≥10 items rated as ‘yes’ for case-control studies = little or no risk of bias; results unlikely to be changed by further research)
- Acceptable quality^+^ (6–11 items rated as ‘yes’ for cohort studies and 5–9 items rated as ‘yes’ for case-control studies = most criteria met; some flaws in the study with an associated risk of bias; conclusions may change in the light of further studies)
- Low quality ^0^ (0–5 items rated as ‘yes’ for cohort studies and 0–4 items rated as ‘yes’ for case-control studies = either most criteria not met, or significant flaws relating to key aspects of study design; conclusions likely to change in the light of further studies).

The quality appraisals will be cross-checked, and any disagreements will be resolved by a consensus-based discussion or through a third party, if necessary. The overall and individual item-specific quality assessment ratings for each study will be presented in Appendix 4 (Table 1 [cohort studies] and Table 2 [case-control studies]).

### Data analysis and synthesis

The collected evidence (study, participant, and outcome characteristics) will be narratively synthesised, appraised, and organised in summary tables and text. The evidence for incidence (global, national, and regional) and subgroups/risk factors in relation to the occurrence of sepsis will be presented separately. Where possible, the community-onset sepsis studies will be stratified by those of healthcare-associated and community-acquired sepsis.

The incidence and/or effect estimates with 95 % confidence intervals (95 % CIs) will be ascertained and presented in tables and figures separately for sepsis, severe sepsis, and septic shock (i.e. forest plots) as available. These outcomes will be further stratified by age, sex, study setting (e.g. hospital ward, nursing care facility, intensive-care unit), and other important characteristics, if data permits. Evidence on risk factors will be summarised separately for case-control and cohort studies.

The study results will be meta-analysed if sufficient degree of similarity exists across characteristics of populations, definitions of exposure and case-control groups, and types of outcomes of sepsis occurrence. The estimates of summary dichotomous outcome measures (e.g. risk or odds ratios) will be pooled using a DerSimonian and Laird random-effects model (no rare events, >10.0 %), Mantel-Haenszel fixed-effects model (low event rates, 5.0 %–10.0 %), or Peto fixed-effects model (very low event rates, < 5.0 % or zero events) [52]. The choice of random-effects model is based on the expectation that there will be clinical and methodological diversity across included studies.

The visual inspection of the forest plots and statistical parameters (Chi-square based *p* value <0.10; *I*^2^ > 50 %) will be used to judge the extent of statistical heterogeneity across the estimates of pooled studies. The heterogeneity will be explored through subgroup analysis (e.g. age, sex, underlying comorbidity) and sensitivity analysis (by study design, summary quality/risk of bias rating, study setting).

The extent of publication bias will be examined if the number of studies reporting quantitative measure(s) of the association between a risk factor and sepsis occurrence is sufficient for the inspection of funnel plot asymmetry [53].

### Rating overall quality of evidence

The overall quality of the body of evidence on risk factors and frequency measures of sepsis occurrence will not be graded because there is no formal and validated grading system directly applicable to the type of evidence synthesised in this review. The widely accepted and used system suggested by the Grading of Recommendations, Assessment, Development, and Evaluation (GRADE) Working Group is ideally applicable to grading the quality of evidence, i.e. multiple patient-oriented outcomes across studies evaluating and comparing different health care interventions [54] and diagnostic accuracy of tests [55].

## Discussion

This systematic review will identify and summarise the relevant evidence on the burden of community-onset sepsis in terms of incidence and risk factors. Major findings of this systematic review will be summarised in conjunction with the study methodological quality. Strengths and limitations (e.g. exclusion of non-English studies, conference abstracts) of the review will be discussed and gaps in the evidence will also be highlighted. The findings of this review and those of other similar reviews will be compared (if identified) for the degree of consistency.

Timeliness and accuracy of diagnosis of sepsis are both crucial aspects for improving the patient’s outcome. The findings will be discussed with a view to better informing PHE recommendations on public-facing campaigns to improve timely presentation and diagnosis of sepsis in the community as well as contribute more widely as a basis to future research and policy on improving public health planning.

## Figures and Tables

**Fig. 1:**
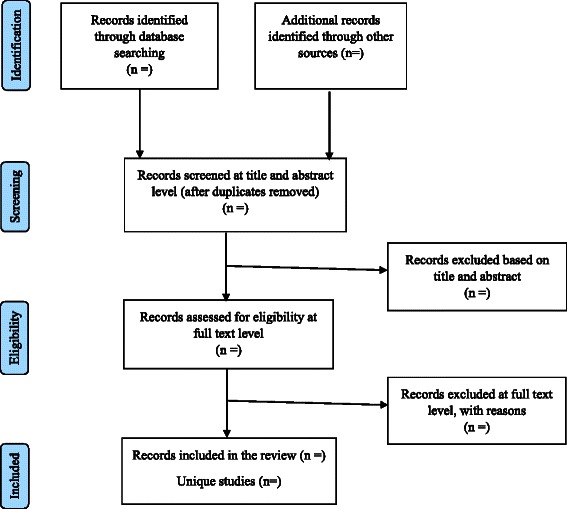
PRISMA study flow diagram

## Appendix 1

### Search strategy for Ovid MEDLINE

1. exp Sepsis/
2. (sepsis or septic?emi* or bact?eremi* or disseminated candidiasis or fung?emi* or septic shock).tw.
3. 1 or 2
4. ep.fs.
5. incidence/
6. exp risk/
7. exp Population Surveillance/
8. exp epidemiologic studies/
9. exp odds ratio/or exp risk/
10. exp Socioeconomic Factors/
11. exp Ethnic Groups/
12. (epidemiology or incidence or risk or mortality or burden or odds ratio or prevalence).tw.
13. 4 or 5 or 6 or 7 or 8 or 9 or 10 or 11 or 12
14. 3 and 13
15. ((community or population) adj2 (onset or acqui* or based)).tw.
16. long term care facilit*.tw.
17. (nursing home* or care home*).tw.
18. exp Residential Facilities/
19. (prehospital or pre-hospital).tw.
20. out-of-hospital.tw.
21. (sepsis related hospitalization or sepsis related hospitalisation).tw.
22. (hospitalised with sepsis or hospitalized with sepsis).tw.
23. admitted with sepsis.tw.
24. 15 or 16 or 17 or 18 or 19 or 20 or 21 or 22 or 23
25. 14 and 24
26. limit 25 to (english language and yr = “2002 -Current”)
27. (comment or letter or editorial).pt.
28. 26 not 27

## Appendix 2

ᅟ

## Appendix 3

### Data extraction sheet for included primary study reports

ᅟ

## Appendix 4

### Methodological quality of included studies

ᅟ

**Table 1:** Methodological quality (risk of bias) in cohort studies (16 items)

Definition (Item #)	Study 1 {#X}	Study 2 {#X}	Study 3 {#X}	Study 4 {#X}	Study 5 {#X}	Study 6 {#X}	Study 7 {#X}
Internal validity							
The study addresses an appropriate and clearly focused question (Item 1)							
Selection of subjects							
The two groups being studied are selected from source populations that are comparable in all respects other than the factor under investigation (Item 2)							
The study indicates how many of the people asked to take part did so, in each of the groups being studied (Item 3)							
The likelihood that some eligible subjects might have the outcome at the time of enrolment is assessed and taken into account in the analysis (Item 4)							
What percentage of individuals or clusters recruited into each arm of the study dropped out before the study was completed (Item 5)							
Comparison is made between full participants and those lost to follow-up, by exposure status (Item 6)							
Assessment							
The outcomes are clearly defined (Item 7)							
The assessment of outcome is made blind to exposure status. If the study is retrospective this may not be applicable (Item 8)							
Where blinding was not possible, there is some recognition that knowledge of exposure status could have influenced the assessment of outcome (Item 9)							
The method of assessment of exposure is reliable (Item 10)							
Evidence from other sources is used to demonstrate that the method of outcome assessment is valid and reliable (Item 11)							
Exposure level or prognostic factor is assessed more than once (Item 12)							
Confounding							
The main potential confounders are identified and taken into account in the design and analysis (Item 13)							
Statistical analysis							
Have confidence intervals been provided? (Item 14)							
Overall assessment of the study							
Taking into account clinical considerations, your evaluation of the methodology used, and the statistical power of the study, do you think there is clear evidence of an association between exposure and outcome? (Item 15)							
Are the results of this study directly applicable to the patient group targeted in this guideline? (Item 16)							
Summary quality (risk of bias) rating							

Possible responses to each item: yes, no, can’t say, or doesn’t apply

≥12 items rated as ‘yes’ - high quality^++^ (little or no risk of bias; results unlikely to be changed by further research)

6–11 items rated as ‘yes’ - acceptable quality^+^ (most criteria met; some flaws in the study with an associated risk of bias; conclusions may change in the light of further studies)

0–5 items rated as ‘yes’ - low quality ^0^ (either most criteria not met, or significant flaws relating to key aspects of study design; conclusions likely to change in the light of further studies)

**Table 2:** Methodological quality (risk of bias) in case-control studies (13 items)

Definition (Item #)	Study 1 {#X}	Study 2 {#X}	Study 3 {#X}	Study 4 {#X}	Study 5 {#X}	Study 6 {#X}	Study 7 {#X}
Internal validity							
The study addresses an appropriate and clearly focused question (Item 1)							
Selection of subjects							
The cases and controls are taken from comparable populations (Item 2)							
The same exclusion criteria are used for both cases and controls (Item 3)							
What percentage of each group (cases and controls) participated in the study? (Item 4)							
Comparison is made between participants and non-participants to establish their similarities or differences (Item 5)							
Cases are clearly defined and differentiated from controls (Item 6)							
It is clearly established that controls are non-cases (Item 7)							
Assessment							
Measures will have been taken to prevent knowledge of primary exposure influencing case ascertainment (Item 8)							
Exposure status is measured in a standard, valid and reliable way (Item 9)							
Confounding							
The main potential confounders are identified and taken into account in the design and analysis (Item 10)							
Statistical analysis							
Confidence intervals are provided (Item 11)							
Overall assessment of the study							
Taking into account clinical considerations, your evaluation of the methodology used, and the statistical power of the study, do you think there is clear evidence of an association between exposure and outcome? (Item 12)							
Are the results of this study directly applicable to the patient group targeted by this guideline? (Item 13)							
Summary quality (risk of bias) rating							

Possible responses to each item: yes, no, can’t say, or doesn’t apply

≥10 items rated as ‘yes’ - high quality^++^ (little or no risk of bias; results unlikely to be changed by further research)

5–9 items rated as ‘yes’ - acceptable quality^+^ (most criteria met; some flaws in the study with an associated risk of bias; conclusions may change in the light of further studies)

0–4 items rated as ‘yes’ - low quality ^0^ (either most criteria not met, or significant flaws relating to key aspects of study design; conclusions likely to change in the light of further studies)

## Abbreviations

ACCP: American College of Chest Physicians
HIV: human immunodeficiency virus
ICD-10: International Classification of Diseases 10th revision
ICU: intensive-care unit
PHE: Public Health England
PIRO: Predisposition, Infection, Response, and Organ Dysfunction
SCCM: Society of Critical Care Medicine
SIRS: Systemic Inflammatory Response Syndrome
UK: United Kingdom
US: United States
